# Knock knock, who’s there?: marine invertebrates in tubes of Ceriantharia (Cnidaria: Anthozoa)

**DOI:** 10.3897/BDJ.8.e47019

**Published:** 2020-01-08

**Authors:** Hellen Ceriello, Celine S.S. Lopes, James Davis Reimer, Torkild Bakken, Marcelo V. Fukuda, Carlo Magenta Cunha, Sérgio N. Stampar

**Affiliations:** 1 Universidade Estadual Paulista "Júlio de Mesquita Filho" (UNESP), FCL, Assis, Brazil Universidade Estadual Paulista "Júlio de Mesquita Filho" (UNESP), FCL Assis Brazil; 2 Universidade Estadual Paulista "Júlio de Mesquita Filho" (UNESP), Instituto de Biociências, Botucatu, Brazil Universidade Estadual Paulista "Júlio de Mesquita Filho" (UNESP), Instituto de Biociências Botucatu Brazil; 3 University of the Ryukyus, Nishihara, Okinawa, Japan University of the Ryukyus Nishihara, Okinawa Japan; 4 Norwegian University of Science and Technology, NTNU University Museum, Trondheim, Norway Norwegian University of Science and Technology, NTNU University Museum Trondheim Norway; 5 Museu de Zoologia da Universidade de São Paulo (MZSP), São Paulo, Brazil Museu de Zoologia da Universidade de São Paulo (MZSP) São Paulo Brazil; 6 Universidade Federal de São Paulo (Unifesp), Instituto do Mar, Santos, Brazil Universidade Federal de São Paulo (Unifesp), Instituto do Mar Santos Brazil

**Keywords:** Biodiversity, Crustacea, Hotspots, Mollusca, Polychaeta, Tube-dwelling anemones.

## Abstract

This study reports on the fauna found in/on tubes of 10 species of Ceriantharia and discusses the characteristics of these occurrences, as well as the use of mollusc shells in ceriantharian tube construction. A total of 22 tubes of Ceriantharia from Argentina, Brazil, Japan, Norway, Portugal and the United States were analysed, revealing 58 species of marine invertebrates using them as alternative substrates. Based on a literature review and analyses of the sampled material, we report new occurrences for *Photis sarae* (Crustacea), *Microgaza rotella* (Mollusca), *Brada* sp., *Dipolydora* spp., *Notocirrus* spp., and *Syllis garciai* (Annelida). The use of mollusc shells in tube construction increases the tubes’ structural resistance and strength. Ceriantharian tubes are suitable alternative substrates for the dwelling of numerous tubicolous and infaunal species that usually burrow into sediments or anchor on fixed or mobile habitats seeking shelter, thus playing a relevant role as local biodiversity hotspots.

## Introduction

Benthic organisms are well adapted to the habitat conditions present in the locations where they live and estimates of abundance of these organisms are usually related to the habitat in which they are found (Hutchings 1998). Moreover, some species require anchoring sites to settle and complete part of or their whole life cycles (Koehl 1984, Chase et al. 2016, Cowan et al. 2016, Ritson-Williams et al. 2016). Thus, the lack of consolidated structures on unconsolidated bottoms leads many benthic settlers to seek different suitable substrates (Betti et al. 2017), amongst which are artificial substrates such as ship hulls (Carraro 2012) or offshore platforms (Bomkamp et al. 2004), and natural substrates, such as marine invertebrate shells (Farrapeira and Calado 2010), corals (Buhl-Mortensen et al. 2010), and ceriantharian tubes.

Ceriantharians (Cnidaria: Anthozoa) are tube-dwelling animals that synthesize their tubes primarily with the use of ptychocysts, a type of cnida only found in this group, combined with small sediment fragments from the sea bottoms where the tube is built (Stampar et al. 2015). The soft texture of ceriantharian tubes would initially appear not to be an attractive feature for the anchoring of invertebrate species that usually use rigid structures as anchoring locations. However, a few studies have reported on species able to settle on this microhabitat (O’Connor et al. 1977,Tiffon 1987, Moore and Cameron 1999, Stampar et al. 2010, Kim and Huys 2012, Goto et al. 2012). In spite of it, the sampling of Ceriantharia is rather troublesome and rare, and tubes are usually overlooked and rarely collected along with polyps, contributing to lack of information about this subject. Thus, the present study reports on invertebrate communities inhabiting tubes of different ceriantharian species from different locations, and discusses their main characteristics.

## Material and methods

### Sampled material

We sampled 22 tubes of 10 species of Ceriantharia by SCUBA surveys in Argentina, Brazil, Japan, Norway, Portugal, and the United States (Table 1). All material, except for *Isarachnanthus nocturnus* den Hartog, 1977 and *Ceriantheomorphe* sp., was preserved along with their polyps and, before analyses, all polyps were removed from their tubes which were kept individually in labelled jars containing 70% ethanol.

### Morphological analyses

Each tube was analyzed separately under a stereomicroscope in a bowl with dark craft foam in the bottom and full of freshwater. All tubes were longitudinally cut with surgical carbon steel scalpels, opened, and fixed in the craft foam using acupuncture needles. Both inner and outer walls were analyzed.

The fauna found in or on the tubes was removed, photographed, and measured using a Zeiss AxioCam MRc5 and Zeiss AxioVision SE64 Rel 4.8 imaging software. Afterwards, the associated fauna was morphologically identified with specific taxonomic keys for each group (see Suppl. material 1).

### Deposit of specimens

Molluscs (shells), polychaetes and peracaridan crustaceans in this study are deposited in the Museum of Zoology of the University of São Paulo (MZSP), NTNU University Museum, Norwegian University of Science and Technology, Trondheim (NTNU-VM), and the Museum of Zoology, University of Campinas (UNICAMP) – (ZUEC).

Ceriantharians were deposited in the American Museum of Natural History (AMNH), National Museum of Rio de Janeiro Federal University (MNRJ), Biology Institute of Rio de Janeiro Federal University (UFRJ Biologia), NTNU-VM, and MZSP.

## Results

A total of 58 species (8 crustaceans, 24 molluscs, 26 polychaetes) was observed in/on ceriantharian tubes (Table 2). It is noteworthy that, although crustaceans and polychaetes in this study were alive at the time of sampling, they were not alive during tube analyzes. The results were separated by taxonomic groups as follows:

### Mollusca

38 mollusc shell specimens, including Gastropoda and Bivalvia (Fig. 1a), were observed and were always found adhered to the outside of the tubes, and none had a periostracum coating, indicating that they were not alive at the time of collection.

#### Gastropods

We observed shells of *Schwartziella bryerea* Montagu, 1803 and *Turbonilla* sp. adhered to the fragile tube of *Arachnanthus* sp., as well as amongst sediments that surrounded the tube. Shells of *Cerithidea balteata* A. Adams, 1855, *Eulima* sp., *Liotella* sp., *Emarginula* sp., *Chrysallida* sp. and *Collonista rubricincta* Mighels, 1845 were found attached to the entire length of the thin and delicate tube of *Isarachnanthus bandanensis* Carlgren, 1924. *Bittiolum varium* Pfeiffer, 1840 was found attached to the tubes of *Isarachnanthus nocturnus*. *Puncturella noachina* Linnaeus, 1771 was, in part, adhered to the thin and fragile tube of *Cerianthus lloydii* Gosse, 1859.

On the tubes of *Ceriantheomorphe brasiliensis* Carlgren, 1931, we noted shells of *B. varium*, *Finella dubia* d'Orbigny, 1840, *Parvanachis obesa* C. B. Adams, 1845, *Bostrycapulus odites* Collin, 2005, *Caecum regulare* Carpenter, 1858 and *Microgaza rotella* Dall, 1881. The tubes of *C. brasiliensis* usually have a high amount of overlap of filaments and, although this pattern was also observed in specimens in this study, no mollusc shells were found between layers, and shells were only found on the outermost surfaces of the tubes.

#### Bivalves

Shells of *Ervilia nitens* Montagu, 1808, *Chama* sp., *Cardites micellus* Penna-Neme, 1971 and *Tivela* sp. were observed adhered on the tube of *Arachnanthus* sp., while *E. nitens*, *Basterotia elliptica* Récluz, 1850 and *Musculus lateralis* Say, 1822 were observed adhered on the tubes of *I. nocturnus*.

Shells of *Sphenia fragilis* H. Adams & A. Adams, 1854, *E. nitens* and *M. lateralis* were observed upon the tubes of *C. brasiliensis*, and shells of *Macomopsis melo* G. B. Sowerby II, 1866 were observed covering considerable areas of the tube of *Ceriantheomorphe* sp.

Different from the tubes above, the only area on the tube of *Ceriantheopsis americana* Agassiz *in* Verrill, 1864 where we observed the presence of mollusc shells, was on its slender end that was vertically inserted into the soft bottom. All specimens observed were *Cumingia lamellosa* G. B. Sowerby I, 1833 and these were found in high amounts and firmly attached to the tube.

### Crustacea (Peracarida)

We observed 29 peracaridans (Fig. 1b A-H), belonging to 8 families, including 5 amphipod species, 2 isopod species and 1 tanaidacean species on the tubes of three ceriantharian species.

Most peracaridans were found in areas far from the ceriantharian tentacles, thus not easily accessible to the ceriantharian. No specimen was found inside the tubes or amongst tube layers. On the tubes of *Ceriantheomorphe brasiliensis*, we observed the amphipods *Ampelisca burkei* J.L. Barnard & Thomas, 1989, *Cymadusa filosa* Savigny, 1816, *Elasmopus pectenicrus* Spence Bate, 1862 and *Photis sarae* Souza-Filho & Serejo, 2010, and the isopod *Paranthura urochroma* Pires, 1981 firmly attached to the tube external wall; both amphipods and isopods were surrounded by ptychocyst filaments. Additionally, we found tanaidaceans of species *Chondrochelia savignyi* Kroyer, 1842; however, those were free from ptychocyst filaments and were not firmly attached. *Monocorophium acherusicum* Costa, 1853 (Amphipoda) and *Idotea balthica* Pallas, 1772 (Isopoda) were also found surrounded by ptychocyst filaments and attached to the external wall of the tube of *Ceriantheopsis lineata* Stampar, Scarabino, Pastorino & Morandini, 2015. One specimen of *P. sarae* was noted amongst algae thalli covering the tube of *Isarachnanthus nocturnus*. It is noteworthy that the amphipod was not directly attached to the tube, but instead it was freely on its surface.

### Annelida (Polychaeta)

A total of 122 polychaetes (Fig. 1b I-L), including 17 families and 26 species, were found in or on tubes of six species of Ceriantharia. Some of the specimens were not possible to identify further than family or genus, as they were fragmented or in poor condition.

We observed one specimen of *Lysilla loveni* Malmgren, 1866 (Terebellidae), two cirratulids, two paraonids and two syllids in between layers of the tube of *Botrucnidifer norvegicus* Carlgren, 1912. On the external wall of the tube of *Ceriantheomorphe brasiliensis*, we found cirratulids (*Cirriformia* spp.), eunicids (*Lysidice* spp.), nereidids (*Neanthes* sp.), syllids (*Exogone* spp., *Myrianida* sp. and *Syllis prolifera* Krohn, 1852), and spionids (*Aonides* sp. and *Dipolydora* spp.), and one specimen each of Sabellidae (*Branchiomma* sp.), Flabelligeridae (*Brada* sp.), Magelonidae (*Magelona* sp.), Polynoidae (*Malmgreniella* sp.), Capitellidae (*Mediomastus* spp.), and Phyllodocidae. Only some specimens had ptychocyst filaments surrounding them and keeping them firmly attached to the tube. We observed *Dipolydora* spp. amongst algae thalli covering this tube, as well as in between folds of layers of the tube of *C. brasiliensis* from Guanabara Bay.

The heavy tubes of *Ceriantheopsis lineata* showed many perforations that were occupied by either deeply or superficially burrowed polychaetes between some layers. Beneath layers, we observed some spionids (*Dipolydora* spp.) and single specimens of capitellid (*Mediomastus* spp.), cirratulid (*Cirriformia* spp.), and oenonid (*Notocirrus* spp.). The removal of layers also revealed empty boring holes under them. Moreover, we found *Syllis garciai* Campoy, 1982 (Syllidae) and one phyllodocid on the tube surface, surrounded by ptychocyst filaments and mucus, respectively.

Some *Parasabella* sp., *Lysidice* spp., *Cirriformia* spp., and *Spirobranchus* sp. were found amongst algae thalli partially covering one of the tubes of *Isarachnanthus nocturnus*. However, they were not attached to the tube and neither had ptychocyst filaments surrounding them. Additionally, we observed *Notocirrus* spp. on the surface of this tube.

We observed one maldanid on the surface of the tube of *Ceriantheomorphe* sp., as well as large *Nereis* sp. partially burrowed, and small groups of *Sternaspis* sp. (3 specimens each group) both superficially anchored and deeply burrowed into tube layers.

Finally, we found 36 *Notocirrus* spp. and two syllids on tubes of *Pachycerianthus schlenzae* Stampar, Morandini & Silveira, 2014, either burrowed between layers or attached to the surface of the tubes. In both cases, there were some specimens coated by their own mucus, but none was firmly attached to the tubes.

## Discussion

There have been some previous studies on the presence of marine invertebrates anchored on ceriantharian tubes, with results suggesting that they are a suitable option as a consolidated structure for the settlement in unconsolidated bottoms (e.g. O’Connor et al. 1977, Moore and Cameron 1999, Stampar et al. 2010, Kim and Huys 2012). Our results not only corroborate the use of ceriantharian tubes as alternative substrates for other organisms, but also indicate a different anchoring method for species of the three phyla evaluated, Mollusca, Arthropoda (Crustacea) and Annelida (Polychaeta). Furthermore, we suggest possible benefits acquired by species on ceriantharian tubes, discuss the use of mollusc shells in ceriantharian tube construction, and report new location records for six taxa.

### Anchoring methods

We did not observe whether peracaridans and polychaetes voluntarily settle on ceriantharian tubes or are incorporated into the tubes by the ceriantharians. In spite of this, our results show that most of these specimens were found in areas of the tubes where the tentacles of the ceriantharian could not easily reach them. Thus, it is most likely that these species have actively recruited this alternative substrate than have been incorporated into it by the actions of the ceriantharian. As we could not evaluate this possibility, this hypothesis cannot be excluded.

Ptychocyst filaments are the most common material in ceriantharian tubes (Stampar et al. 2015). Notably, most amphipods and isopods firmly anchored to ceriantharian tubes were surrounded by filaments (e.g. *A. burkei*, *C. filosa*, *I. balthica*, *M. acherusicum*, *P. urochroma*, and *P. sarae*), while some other specimens, such as *C. savignyi*, were not. Likewise, some polychaetes were observed surrounded by filaments (e.g. *S. garciai)* and thus firmly anchored, while others were coated by their own mucus (e.g. phyllodocids and *Notocirrus* spp.) and only superficially anchored. Stampar et al. (2015) suggested that ptychocyst filaments have adhesive properties and our observations support this suggestion, as it is likely that the adhesive property of ptychocyst filaments is used by peracaridans and polychaetes as an anchoring method to settle on ceriantharian tubes. Otherwise, specimens not surrounded by ptychocyst filaments must have alternative anchoring methods to keep them on tubes.

### Burrowers and tubicolous species in ceriantharian tubes

Crustaceans, tubeworms and ceriantharians often acquire shelter against predators in self-built-tubes which may be rigid, as in some cirratulids, sabellids and serpulids (Fauchald and Jumars 1979, ten Hove and Vandenhurk 1993, Díaz-Castañeda and Reish 2009, Jumars et al. 2015, Silva and Lana 2018).

We observed the polychaetes *Lysidice* spp. anchored on ceriantharian tubes. As members of this genus commonly excavate galleries or temporarily occupy empty galleries/tubes of other organisms (Díaz-Castañeda and Reish 2009), it is possible that *Lysidice* spp. use ceriantharian tubes as alternative habitats.

Tube-dwelling amphipods, isopods, and tanaidaceans usually burrow directly into the soft bottom, forming mucous tubes for habitation (Greve 1967, Johnson and Attramadal 1982, Thistle et al. 1985). For instance, the amphipod *Photis sarae* was observed anchored on tubes of *I. nocturnus* and *C. brasiliensis.* However, this species is usually found in soft tubes built with mucus, small sediments and, sometimes, living organisms (e.g. algae) (Souza-Filho and Serejo 2010), similar to Ceriantharia. We also observed other tube-dwelling peracaridans coated by ptychocyst filaments and attached to the surface of ceriantharian tubes, suggesting that, by using ceriantharian tubes, peracaridans can be sheltered, without the necessity of building their own tubes.

### Role of mollusc shells in tube construction

Mollusk shells were observed on all ceriantharian tubes examined. However, the absence of periostracum coating these shells suggests that ceriantharians do not shelter living molluscs (Meenakshi et al. 1969, Taylor and Kennedy 1969), but instead they adhere empty shells to their tubes, using them as a relevant component for the tube construction. The addition of mollusc shells and other sediment remains as tube constituents may reinforce the tube, increasing its resistance and, thus, having an architectural role. Moreover, the external surfaces of all shells were usually very worn, indicating that they were part of the seafloor sediment rather than part of living assemblages. Although our data do not allow us to assess how the shells were obtained during tube construction, future studies would be useful to provide such information (e.g. is there any special behavior associated with inclusion of mollusc shells?) and to examine if it is possible that ceriantharian tubes shelter living molluscs.

Bürkli and Wilson (2017) have suggested that empty mollusc shells enable the understanding of biodiversity patterns of Mollusca fauna at a specific site and can thus be used to provide data on ecological and evolutionary timescales. Accordingly, a similar role could be attributed to the accumulation of shells in ceriantharian tubes, reflecting the species richness of living molluscs in the surrounding habitat.

### New location reports of molluscs, peracaridans and polychaetes

This is the first record of *Microgaza rotella* (Mollusca) and *Brada* sp. (Polychaeta) in Laje de Santos, and *Photis sarae* (Peracarida) in São Sebastião and Laje de Santos, São Paulo State, in southeastern Brazil. To date, *M. rotella* had been reported as occurring from the southeastern United States to northern Brazil (Rios 2009), and, since that there have been no other records in literature regarding this species in southeastern Brazil *M. rotella* may occur naturally at this location (Laje de Santos) and may be rare or allochthonous (i.e. originated in a region other than where it was found) and transported by other species. *Brada* had been previously reported in Brazil only from Ubatuba City (Amaral et al. 2013), while *P. sarae* had only been previously reported in Rio de Janeiro State (Souza-Filho and Serejo 2010).

This is also the first record of *Dipolydora* in Rio de Janeiro State, and *Notocirrus* spp. and *Syllis garciai* in Espírito Santo State. *Dipolydora* had only been previously reported from Brazil in São Paulo, Paraná and Espírito Santo States. *Notocirrus* had been reported occurring in São Paulo, Rio de Janeiro, Paraná and Bahia States, while *Syllis garciai* had only been previously reported in São Paulo State (Amaral et al. 2013).

It is noteworthy that *Lysilla loveni* (Polychaeta) was found on the tube of a Nordic Ceriantharia species, *Botrucnidifer norvegicus.* This polychaete species has only rarely been found and usually as single occurrences scattered along the Norwegian coast (Holthe 1977, Holthe 1986).

### Tubes of Ceriantharia as anchoring points

Biogenic structures, such as ceriantharian tubes, play a major role in altering community structure, thus affecting species richness and individual abundances (Hoey et al. 2008). Tubes may affect the stability of the sea bottom and provide refugia from predation, as well as surfaces for the recruitment of benthic organisms (Woodin 1978, Woodin 1981). In fact, species abundance and richness have been observed to be greater around or on tubes than in areas without tubes (Callaway 2003, Callaway 2006, Rees et al. 2005). In our study, we did not compare the fauna from ceriantharian tubes to that from the surrounding sea bottoms however, our results demonstrate that ceriantharian tubes appear to be suitable alternative substrates for numerous species, especially tubicolous and infaunal invertebrates that usually spend much energy burrowing into sediments or anchoring on fixed or mobile habitats while seeking shelter. Moreover, other than shelter, residents on and in ceriantharian tubes may also acquire protection. Therefore, the tubes of Ceriantharia may play an important role as small-scale biodiversity hotspots.

## Supplementary Material

5C234997-5224-5E51-9760-ADACD9A0301C 10.3897/BDJ.8.e47019.suppl1 Supplementary material 1 Taxonomic Keys and Material examined Data type: Morphological
Brief description: List of material examined in this study and taxonomic keys used for their identification.
File: oo_356210.doc https://binary.pensoft.net/file/356210 Hellen Ceriello, Celine S. S. Lopes, James D. Reimer, Torkild Bakken, Marcelo V. Fukuda, Carlo M. Cunha, Sérgio N. Stampar

## Figures and Tables

**Figure 1a.**
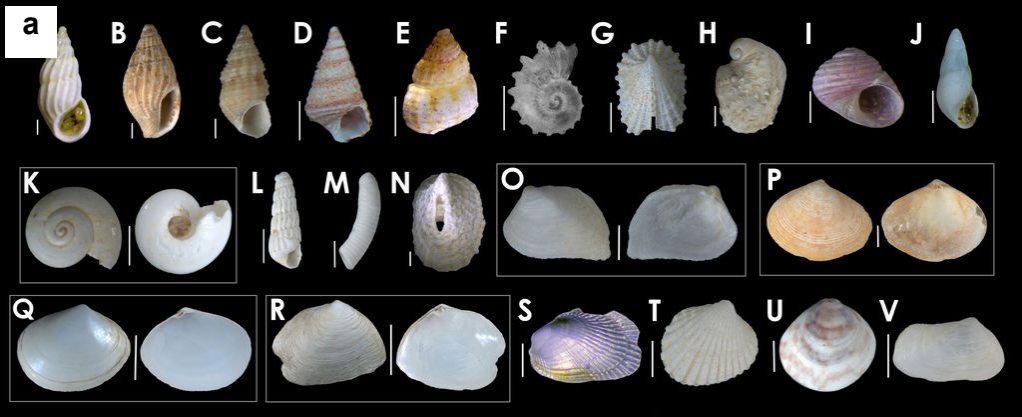
Some Mollusca found on ceriantharian tubes. **(A)** *Schwartziella bryerea* **(B)** *Parvanachis obesa* **(C)** *Bittiolum varium* (**D)** *Cerithidea balteata* **(E)** *Chrysallida* sp. **(F)** *Liotella* sp. **(G)** *Emarginula* sp. **(H)** *Bostrycapulus odites* **(I)** *Collonista rubricincta* **(J)** *Eulima* sp. **(K)** *Microgaza rotella* **(L)** *Turbonilla* sp. **(M)** *Caecum regulare* **(N)** *Puncturella noachina* **(O)** *Basterotia elliptica* **(P)** *Ervilia nitens* **(Q)** *Macomopsis melo* **(R)** *Cumingia lamellosa* **(S)** *Musculus lateralis* **(T)** *Cardites micellus* **(U)** *Tivela* sp. **(V)** *Sphenia fragilis*. Scale bars **(A-N)** 500 µm **(O–U)** 500 µm **(V)** 100 µm.

**Figure 1b.**
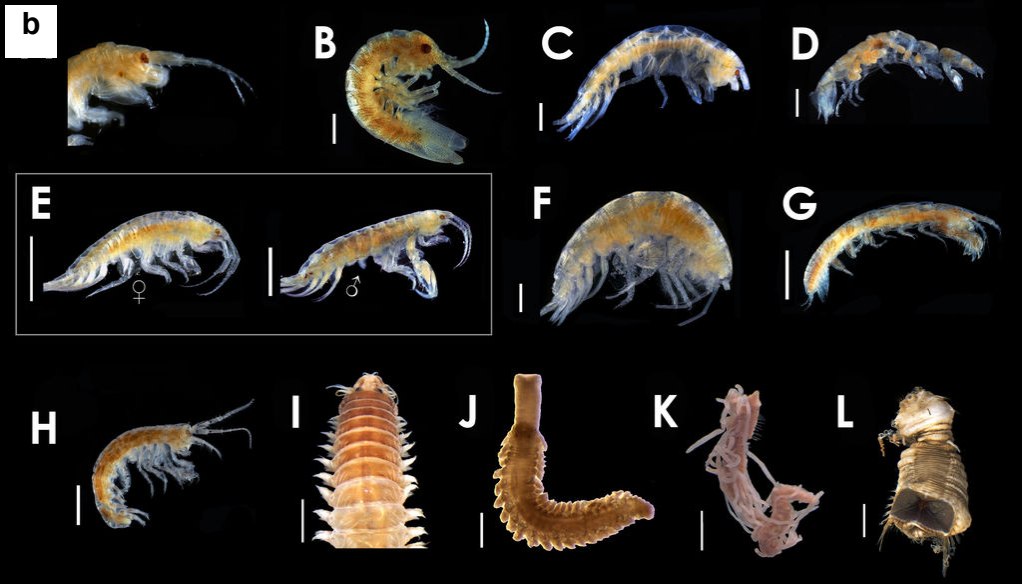
Some Crustacea and Polychaeta found in/on ceriantharian tubes. **(A)** *Monocorophium acherusicum* **(B)** *Idotea balthica* **(C)** *Cymadusa filosa* **(D)** *Paranthura urochroma* **(E)** *Photis sarae*, female and male, respectively **(F)** *Ampelisca burkei* **(G)** *Chondrochelia savignyi* **(H)** *Elasmopus pectenicrus* **(I)** *Nereis* sp. **(J)** Phyllodocidae, indet. **(K)** *Cirriformia* sp. **(L)** *Sternaspis* sp. Scale bars: **(A-H)** 1000 μm **(I)** 2000 μm **(J)** 600 μm **(K)** 1000 μm **(L)** 3000 μm.

**Table 1.** Species of Ceriantharia, for which tubes were investigated in this study, their taxonomic family, number of specimens, and collection sites.

**Species**	**Family**	**Number of specimens**	**Collection sites**
*Arachnanthus* sp.	Arachnactidae	1	Brazil: São Sebastião (São Paulo)
*Botrucnidifer norvegicus*	Botrucnidiferidae	2	Norway: Agdenes, Stadsbygd (Trondheimsfjord)
*Ceriantheomorphe brasiliensis*	Cerianthidae	7	Brazil: Angra dos Reis, Arraial do Cabo, Guanabara Bay (Rio de Janeiro), Canasvieiras (Santa Catarina), São Sebastião, Laje de Santos (São Paulo)
*Ceriantheomorphe* sp.	Cerianthidae	1	Portugal: Aveiro Lagoon (Aveiro)
*Ceriantheopsis americana*	Cerianthidae	1	USA: St. Andrews Bay (Florida)
*Ceriantheopsis lineata*	Cerianthidae	2	Argentina: Port of Quequén (Buenos Aires) Brazil: Vitória (Espírito Santo)
*Cerianthus lloydii*	Cerianthidae	1	Norway: Trondheim
*Isarachnanthus bandanensis*	Arachnactidae	1	Japan: Mizugama (Okinawa)
*Isarachnanthus nocturnus*	Arachnactidae	4	Brazil: Boa Viagem beach (Salvador/Bahia), São Sebastião (São Paulo)
*Pachycerianthus schlenzae*	Cerianthidae	2	Brazil: Guaraparí, Vitória (Espírito Santo), Nova Viçosa (Bahia)

**Table 2.** Taxa and number of specimens found on species of tubes of Ceriantharia.

Taxa found on tubes of Ceriantharia	Tube species of Ceriantharia									
	*Arachnanthus* sp.	*Botrucnidifer norvegicus*	*Ceriantheomorphe brasiliensis*	*Ceriantheomorphe* sp.	*Ceriantheopsis americana*	*Ceriantheopsis lineata*	*Cerianthus lloydii*	*Isarachnanthus bandanensis*	*Isarachnanthus nocturnus*	*Pachycerianthus schlenzae*
Mollusca	*Cardites micellus* (1) *Chama* sp. (3) *Ervilia nitens* (6) *Schwartziella bryerea* (1) specimen *Tivela* sp. (1) *Turbonilla* sp. (1)	-	*Bittiolum varium* (1) *Bostrycapulus odites* (1) *Caecum regulare* (1) *Ervilia nitens* (1) *Finella dubia* (1) *Microgaza rotella* (1) *Musculus lateralis* (1) *Parvanachis obesa* (1) *Sphenia fragilis* (1)	*Macomopsis melo* (3)	*Cumingia lamellosa* (2)	-	*Puncturella noachina* (1)	*Cerithidea balteata* (1) *Chrysallida* sp. (1) *Collonista rubricincta* (1) *Emarginula* sp. (1) *Eulima* sp. (1) *Liotella* sp. (1)	*Basterotia elliptica* (1) *Bittiolum varium* (1) *Ervilia nitens* (1) *Musculus lateralis* (1)	-
Crustacea	-	-	*Ampelisca burkei* (1) *Chondrochelia savignyi* (9) *Cymadusa filosa* (4) *Elasmopus pectenicrus* (1) *Paranthura urochroma* (1) *Photis sarae* (10)	-	-	*Idotea balthica* (1) *Monocorophium acherusicum* (1)	-	-	*Photis sarae* (1)	-
Polychaeta	-	Cirratulidae (2) Paraonidae (2) *Lysilla loveni* (1) Syllidae (2)	*Aonides* sp. (2) *Branchiomma* sp. (1) *Brada* sp. (1) *Cirriformia* sp. (24) *Dipolydora* sp. (4) *Exogone* sp. (1) *Lysidice* sp. (3) *Magelona* sp. (1) *Malmgreniella* sp. (1) *Mediomastus* sp. (1) *Myrianida* sp. (1) *Neanthes* sp. (4) Phyllodocidae (1) *Syllis prolifera* (1)	Maldani1dae (1) *Nereis* sp. (2) *Sternaspis* sp. (6)	-	*Cirriformia* sp. (1) *Dipolydora* sp. (9) *Notocirrus* sp. (1) *Mediomastus* sp. (1) Phyllodocidae (1) *Syllis garciai* (1)	-	-	*Cirriformia* sp. (1) *Lysidice* sp. (2) *Notocirrus* sp. (1) *Parasabella* sp. (3) *Spirobranchus* sp. (1)	*Exogone* sp. (1) *Notocirrus* sp. (36) Syllidae (1)
