# Supplementary material for: Knock knock, who’s there?: marine invertebrates in tubes of Ceriantharia (Cnidaria: Anthozoa)
Source: Biodivers Data J. 2020 Jan 8;8:e47019. doi: 10.3897/BDJ.8.e47019 (PMC6962252; doi:10.3897/BDJ.8.e47019)
Supplement: Supplementary material 1 — Taxonomic Keys and Material examined [file bdj-08-e47019-s001.doc]

**SUPPLEMENTARY MATERIAL**

**Taxonomic keys**

Both the associated fauna and ceriantharian tubes (based on polyps) were morphologically identified with specific taxonomic keys for each group:

Ceriantharia: Carlgren (1912), Spier et al. (2012), Stampar et al. (2012), and Stampar et al. (2016a,b).

Mollusca: Penna (1971), Abbott (1974), Okutani (2000), Rios (2009), and Dornelas and Simone (2011).

Crustacea: Moreira (1972), Pires (1981), Barnard and Thomas (1989), Peart (2004), Valério-Berardo and Wakabara (2006), Souza-Filho et al. (2009), Bamber (2010), and Andrade and Senna (2017).

Polychaeta: Holthe (1986a), Ryland and Hayward (1995), Rouse and Pleijel (2001) and Harris et al. (2009).

**SPECIES RECORDED**

**Mollusca**

Mollusks found on the ceriantharian tubes: *Norway*, Trondheim, on the tube of *Cerianthus lloydii* Gosse, 1859 (coll. 07.viii.1929), NTNU-VM 40513, 1 shell, Fissurellidae, *Puncturella noachina* Linnaeus, 1771. *Portugal*, Aveiro, Aveiro Lagoon, on the tube of *Ceriantheomorphe* sp. (C. Hernandez and technical group of Aveiro University coll. 08.vi.2016), MZSP 136932, 3 shells, Tellinidae, *Macomopsis melo* G.B. Sowerby II, 1866. *USA*, Florida, St. Andrews Bay, on the tube of *Ceriantheopsis americana* Agassiz *in* Verrill, 1864 (AMNH 3451 coll. 23.ix.1993), MZSP 136931, 2 shells, Semelidae, *Cumingia lamellosa* G. B. Sowerby I, 1833. *Japan*, Okinawa, Mizugama, on the tube of *Isarachnanthus bandanensis* Carlgren, 1924 (coll. 07.xi.2016), MZSP 136938, 1 shell, Potamididae, *Cerithidea balteata* A. Adams, 1855; MZSP 136942, 1 shell, Pyramidellidae, *Chrysallida* sp.; MZSP 136940, 1 shell, Skeneidae, *Liotella* sp.; MZSP 136941, 1 shell, Fissurellidae, *Emarginula* sp.; MZSP 136943, 1 shell, Colloniidae, *Collonista rubricincta* Mighels, 1845; MZSP 136939, 1 shell, Eulimidae, *Eulima* sp. *Brazil*, São Paulo, São Sebastião, on the tube of *Arachnanthus* sp. (coll. 21.vii.2015), MZSP 136925, 1 shell, Zebinidae, *Schwartziella bryerea* Montagu, 1803; MZSP 136926, 1 shell, Pyramidellidae, *Turbonilla* sp.; MZSP 136927, 6 shells, *Ervilia nitens* Montagu, 1808; MZSP 136928, 3 shells, Chamidae, *Chama* sp.; MZSP 136929, 1 shell, Carditidae, *Cardites micellus* Penna-Neme, 1971; MZSP 136930, 1 shell, Veneridae, *Tivela* sp.; Rio de Janeiro, Angra dos Reis and Santa Catarina, Canasvieiras, on the tubes of *Ceriantheomorphe brasiliensis* Carlgren, 1931 (MZSP 8475 S. Stampar and A. Morandini coll. 08.vii.2009), MZSP 136948, 1 shell, Columbellidae, *Parvanachis obesa* C. B. Adams, 1845; MZSP 136946, 1 shell, Cerithiidae, *Bittiolum varium* Pfeiffer, 1840; MZSP 136944, 1 shell, Calyptraeidae, *Bostrycapulus odites* Collin, 2005; MZSP 136945, 1 shell, Scaliolidae, *Finella dubia* d'Orbigny, 1840; MZSP 136947, 1 shell, Myidae, *Sphenia fragilis* H. Adams & A. Adams, 1854; São Paulo, Laje de Santos, on the tube of *C. brasiliensis* (S. Stampar and G. Kodja coll. 22.vii.2007), MZSP 136950, 1 shell, Solariellidae, *Microgaza rotella* Dall, 1881; MZSP 136952, 1 shell, Caecidae, *Caecum regulare* Carpenter, 1858; MZSP 136949, 1 shell, *E. nitens*; MZSP 136951, 1 shell, *Musculus lateralis* Say, 1822; São Paulo, São Sebastião, on the tube of *Isarachnanthus nocturnus* (S. Stampar and A. Morandini coll. 16.ix.2011), MZSP 136953, 1 shell, *B. varium*; Bahia, Salvador, Boa Viagem Beach, on the tube of *I. nocturnus* (coll. 05.iv.2009), MZSP 136935, 1 shell, Basterotiidae, *Basterotia elliptica* Récluz, 1850; MZSP 136936, 1 shell, Semelidae, *E. nitens*; MZSP 136937, 1 shell, Mytilidae, *M. lateralis*.

**Crustacea (Peracarida)**

Peracaridans found on ceriantharian tubes: *Argentina*, Port of Quequén, on the tube of *Ceriantheopsis lineata* Stampar et al., 2015 (in Stampar et al. 2016b) (MZSP 2686 G. Pastorino coll. 10.xii.2008), ZUEC 4145, 1 specimen, Corophiidae, *Monocorophium* *acherusicum* Costa, 1853; ZUEC 4152, 1 specimen, Idoteidae, *Idotea balthica* Pallas, 1772. *Brazil*, Rio de Janeiro, Angra dos Reis and Arraial do Cabo, on the tubes of *Ceriantheomorphe brasiliensis* (MNRJ 2766 and MZSP 8470 coll. 07.xii.1993), ZUEC 4151, 1 specimen, Ampithoidae, *Cymadusa filosa* Savigny, 1816; ZUEC 4154, 1 specimen, Paranthuridae, *Paranthura urochroma* Pires, 1981; ZUEC 4143, 4 specimens, Photidae, *Photis sarae* Souza-Filho & Serejo, 2010; São Paulo, Laje de Santos, on the tubes of *C. brasiliensis* (S. Stampar and G. Kodja coll. 22.vii.2007), ZUEC 4150, 1 specimen, Ampeliscidae, *Ampelisca burkei* J.L. Barnard & Thomas, 1989; ZUEC 4142, 6 specimens, Photidae, *P. sarae;* São Paulo, São Sebastião, on the tubes of *C. brasiliensis* (MZSP 8471 S. Stampar and A. Morandini coll. 08.vii.2009), ZUEC 4147 and ZUEC 4148, 9 specimens, Leptocheliidae, *Chondrochelia savignyi* Krøyer, 1842; ZUEC 4149, 3 specimens, Ampithoidae, *C. filosa*; ZUEC 4153, 1 specimen, Maeridae, *Elasmopus pectenicrus* Bate, 1862; on the tube of *Isarachnanthus nocturnus* (S. Stampar and A. Morandini coll. 16.ix.2011), ZUEC 4144, 1 specimen, Photidae, *P. sarae*.

**Annelida (Polychaeta)**

Polychaetes found on ceriantharian tubes: *Argentina*, Port of Quequén, on the tube of *Ceriantheopsis lineata* (MZSP 2686 G. Pastorino coll. 10.xii.2008), MZSP 3549, 1 specimen, Phyllodocidae Örsted, 1843; MZSP 3550, 9 specimens, Spionidae, *Dipolydora* spp. *Brazil*, Espírito Santo, on the tube of *C. lineata,* MZSP 3553, 1 specimen, Oenonidae, *Notocirrus* spp.; MZSP 3554, 1 specimen, Syllidae, *Syllis garciai* Campoy, 1982; Rio de Janeiro, Angra dos Reis, on the tube of *Ceriantheomorphe brasiliensis* (MNRJ 2766 coll. 07.xii.1993), MZSP 3535, 1 specimen, Phyllodocidae; Arraial do Cabo, on the tube of *C. brasiliensis*, MZSP 3536, 1 specimen, Syllidae, *Myrianida* sp.; Guanabara Bay, on the tube of *C. brasiliensis* (UFRJ Biologia 2-141)*,* MZSP 3537, 4 specimens, Spionidae, *Dipolydora* spp.; São Paulo, São Sebastião, on the tubes of *C. brasiliensis* (MZSP 8471 S. Stampar and A. Morandini coll. 08.vii.2009), MZSP 3538, 1 specimen, Capitellidae, *Mediomastus* spp.; MZSP 3539, 24 specimens, Cirratulidae, *Cirriformia* spp.; MZSP 3540, 3 specimens, Eunicidae, *Lysidice* spp.; MZSP 3541, 1 specimen, Magelonidae, *Magelona* sp.; MZSP 3542, 4 specimens, Nereididae, *Neanthes* sp.; MZSP 3543, 1 specimen, Sabellidae, *Branchiomma* sp.; MZSP 3544, 1 specimen, Syllidae, *Exogone* spp.; MZSP 3545, 1 specimen, Polynoidae, *Malmgreniella* sp.; MZSP 3546, 2 specimens, Spionidae, *Aonides* sp.; on the tubes of *Isarachnanthus nocturnus* (S. Stampar and A. Morandini coll. 16.ix.2011), MZSP 3556, 1 specimen, Cirratulidae, *Cirriformia* spp.; MZSP 3557, MZSP 3560, 2 specimens, Eunicidae, *Lysidice* spp.; MZSP 3558, 3 specimens, Sabellidae, *Parasabella* sp.; MZSP 3559, 1 specimen, Serpulidae, *Spirobranchus* sp.; Laje de Santos, on the tube of *C. brasiliensis* (S. Stampar and G. Kodja coll. 22.vii.2007), MZSP 3547, 1 specimen, Flabelligeridae, *Brada* sp.; MZSP 3548, 1 specimen, Syllidae, *Syllis prolifera* Krohn, 1852; Salvador, Boa Viagem Beach, on the tube of *I. nocturnus* (coll. 05.iv.2009), MZSP 3555, 1 specimen, Oenonidae, *Notocirrus* spp.; Espírito Santo, Guaraparí, on the tube of *Pachycerianthus schlenzae* Stampar et al., 2014 (MZSP 1949 coll. 12.x.2008), MZSP 3561, 27 specimens, Oenonidae, *Notocirrus* spp.; Bahia, Nova Viçosa, on the tube of *P. schlenzae* (MNRJ 2852 coll. 20.x.2011), MZSP 3562, 9 specimens, Oenonidae, *Notocirrus* spp.; 1 specimen, Syllidae; MZSP 3563, 1 specimen, Syllidae, *Exogone* spp. *Norway*, Trondheim, Agdenes, on the tube of *Botrucnidifer norvegicus* Carlgren, 1912 (coll. 07.ix.1927), NTNU-VM 40494, 2 specimens, Cirratulidae Ryckholt, 1851; Stadsbygd, on the tube of *B. norvegicus* (coll. 28.vii.1926), NTNU-VM 40515, 1 specimen, Terebellidae, *Lysilla loveni* Malmgren, 1866; NTNU-VM 40515, 2 specimens, Paraonidae Cerruti, 1909; NTNU-VM 40515, 2 specimens, Syllidae Grube, 1850. *Portugal*, Aveiro, Aveiro Lagoon, on the tube of *Ceriantheomorphe* sp. (C. Hernandez and technical group of Aveiro University coll. 08.vi.2016), MZSP 3564, 1 specimen, Maldanidae Malmgren, 1867; MZSP 3565, 2 specimens, Nereididae, *Nereis* sp.; MZSP 3566, 6 specimens, Sternaspidae, *Sternaspis* sp.

**REFERENCES**

Abbott, R.T. (1974). American Seashells; The marine molluska of the Atlantic and Pacific coasts of North America, second ed. Van Nostrand Reinhold.

Andrade, L.F., and Senna, A.R. (2017). **Four new species of**Cymadusa**Savigny, 1816 (Amphipoda: Ampithoidae) and new records of**C. filosa**Savigny, 1816 from Brazilian coast. Zootaxa 4226, 359**–**389.** doi: 10.11646/zootaxa.4226.3.3

Bamber, R.N. (2010). In the footsteps of Henrik Nikolaj Krøyer: the rediscovery and redescription of *Leptochelia savignyi* (Krøyer, 1842) sensu stricto (Crustacea: Tanaidacea: Leptocheliidae). Proc. Biol. Soc. Wash. 123, 289–311. doi: 10.2988/10-14.1

Barnard, J.L., and Thomas, J.D. (1989). A new species, *Ampelisca burkei*, (Crustacea, Amphipoda) from Florida. Proc. Biol. Soc. Was. 102, 375–384.

Carlgren, O. (1912). Ceriantharia. Dan Ingolf-Exp. 5, 1–79.

Dornelas, A.P.S., and Simone, L.R.L. (2011). Annotated list of type specimens of mollusks deposited in Museu de Zoologia da Universidade de São Paulo, Brazil. Arquivos de Zoologia (São Paulo) 42, 1–81. doi: 10.11606/issn.2176-7793.v42i1p1-81

Harris, L.H., de León-González, J.Á., and Salazar-Vallejo, S.I. (2009). Morfología, métodos, clave para familias y classificación, in Poliquetos (Annelida: Polychaeta) de México y América Tropical, parte I., eds. J.Á. de León-González et al. (México, Universidad Autónoma de Nuevo León), 3–32.

Holthe, T. (1986). Evolution, systematics, and distribution of the Polychaeta Terebellomorpha, with a catalogue of the taxa and a bibliography. Gunneria 55, 1–236.

Moreira, P.S. (1972). Species of marine isopoda (Crustacea, Peracarida) from southern Brazil. Bolm. Inst. Oceanogr. 21, 163–179.

Okutani, T. (2000). Marine mollusks in Japan, second ed. Japan, Tokai University Press.

Peart, R.A. (2004). A revision of the *Cymadusa filosa* complex (Crustacea: Amphipoda: Corophioidea: Ampithoidae). J. Nat. Hist. 38, 301–336. doi: 10.1080/0022293021000055441.

Penna, L. (1971). Novas espécies e registros de Limidae e Cartditidae (Pelecypoda) no litoral brasileiro. Pap. Avulsos Zool. 24, 155–159.

Pires, A.M.S. (1981). Anthurids from intertidal and shallow infralittoral waters from southeastern Brazil (Isopoda: Anthuridea). J. Crustacean Biol. 1, 211–226.

Rios, E. (2009). Compendium of Brazilian sea shells. Rio Grande, Editora Evangraf.

Rouse, G.W., and Pleijel, F. (2001). Polychaetes. Oxford, University Press.

Ryland, J., and Hayward, P. (1995). Handbook of the marine fauna of North-West Europe. Oxford, Oxford University Press. X.

Souza-Filho, J.F., Souza, A.M.T., and Valério-Berardo, M.T. (2009). Description of four new species of the genus *Ampelisca* (Amphipoda, Ampeliscidae) from the northeastern and southeastern coasts of Brazil and designation of a neotype for *Ampelisca soleata* Oliveira, 1954. J. Nat. Hist. 43, 2391–2423*.* doi: 10.1080/00222930903100543

Spier, D., Stampar, S.N., and Prantoni, A.L. (2012). New record of the endangered cerianthid *Ceriantheomorphe brasiliensis* (Cnidaria: Hexacorallia) in Paranaguá Bay, southern Brazil. Mar. Biodivers. Rec. 5, e119.

Stampar, S.N., Maronna, M.M., Kitahara, M.V., Reimer, J.D., Beneti, J.S., and Morandini, A.C. (2016a). Ceriantharia in current systematics: life cycles, morphology and genetics, in The Cnidaria, Past, Present and Future, eds. S. Goffredo, Z. Dubinsky (Switzerland, Springer), 61–72. doi: 10.1007/978-3-319-31305-4_5

Stampar, S.N., Maronna, M.M., Vermeij, M.J., Lang da Silveira, F., and Morandini, A.C. (2012). Evolutionary diversification of banded tube-dwelling anemones (Cnidaria; Ceriantharia; *Isarachnanthus*) in the Atlantic Ocean. PLoS One 7, e41091. doi: 10.1371/journal.pone.0041091

Stampar, S.N., Scarabino, F., Pastorino, G., and Morandini, A.C. (2016b) A new species of tube-dwelling anemone (Cnidaria, Anthozoa, Ceriantharia, *Ceriantheopsis*) from the Warm Temperate South-western Atlantic. J. Mar. Biol. Assoc. U.K. 96, 1475–1481. doi: 10.1017/S0025315415001745

Valério-Berardo, M.T., and Wakabara, Y. (2006). Ampeliscidae from the Brazilian coast. Record of *Ampelisca burkey* Barnard & Thomas, 1989 and descriptions of two new species of *Ampelisca* (Crustacea, Amphipoda). Zootaxa 1286, 1–14.
